# Virtual Reality-Based Postural Balance Training in Autistic Children: A Pilot Randomized Controlled Trial

**DOI:** 10.3390/jcm14165616

**Published:** 2025-08-08

**Authors:** Anna Falivene, Gaia Scaccabarozzi, Silvia Busti Ceccarelli, Massimo Molteni, Katrijn Klingels, Evi Verbecque, Fabio Alexander Storm, Emilia Biffi, Alessandro Crippa

**Affiliations:** 1 Scientific Institute IRCCS E. Medea, 23842 Bosisio Parini, Italy; anna.falivene@lanostrafamiglia.it (A.F.); gaia.scaccabarozzi@lanostrafamiglia.it (G.S.); silvia.busti@lanostrafamiglia.it (S.B.C.); massimo.molteni@lanostrafamiglia.it (M.M.); fabio.storm@lanostrafamiglia.it (F.A.S.); alessandro.crippa@lanostrafamiglia.it (A.C.); 2 Rehabilitation Research Centre (REVAL), Faculty of Rehabilitation Sciences, Hasselt University, 3590 Diepenbeek, Belgium; katrijn.klingels@uhasselt.be (K.K.); evi.verbecque@uhasselt.be (E.V.)

**Keywords:** autism spectrum disorder, postural balance, training, immersive virtual reality

## Abstract

**Background/Objectives**: Beyond the core characteristics of the condition, autistic individuals often significantly struggle with postural balance. This pilot study aimed to investigate the effects of an immersive virtual reality-based training administered with Gait Real-time Analysis Interactive Lab (GRAIL) on postural balance of autistic children. **Methods**: A total of 20 autistic participants aged 6 to 13 were enrolled in a 5-week randomized, parallel-group, open-label, controlled trial, and received either balance training with the GRAIL system or no training. The trial was registered at ClinicalTrials.gov (identifier: NCT04276571). The primary outcome measures were the change in center of pressure (CoP) metrics during GRAIL balance assessments and the change in motor skills as assessed with Movement Assessment Battery for Children-2. Secondary outcome measures included parent-report Developmental Coordination Disorder Questionnaire, center of mass metrics, and gait parameters evaluated with GRAIL. ANCOVA tests were performed for all outcomes, with time (T0 and T1) as within-subjects factor, the group (training and control groups) as between-subjects factor, and considering age as covariate. **Results:** Slight but significant time by group interactions were found in some CoP metrics (i.e., sway path length, velocity in the antero-posterior direction, and the jerk). **Conclusions:** These findings preliminarily suggest that a virtual reality-based training may induce slight modifications in postural balance strategies, which can be enhanced with longer or more intensive training.

## 1. Introduction

Autism spectrum disorder (henceforth, autism) is a highly heterogeneous neurodevelopmental condition, clinically defined by persistent social communication difficulties and patterns of restricted repetitive behaviors or interests [1]. Beyond these core characteristics, autistic individuals often struggle with performing motor activities, including balance tasks [2,3,4]. Adequate postural balance is essential for early typical motor development [5], ensuring control of posture and coping with destabilizing forces [6,7].

Postural balance difficulties in autism have been found to be significantly associated with the degree of socio-communicative atypicalities [8,9] and appear to persist into adulthood [10]. Moreover, autistic individuals experience differences in processing sensory information from the visual, vestibular, and proprioceptive systems, which may, in turn, affect postural balance [2,10].

Previous research has proposed different strategies for interventions that help children cope with postural balance difficulties in autism, such as animal-assisted therapies, martial arts, aquatic exercises, virtual reality-based games, and recreational activity-based interventions (e.g., dance, skating) (for a recent systematic review, see [11]). In recent years, a number of studies have focused on the potential of virtual reality (VR) as an innovative intervention approach to enhance patients’ engagement and motivation. VR environments allow the user to interact with a synthetic world through multiple sensory channels (i.e., visual, auditory, and haptic-sense of touch), experiencing different degrees of immersion [12]. Thus, VR in rehabilitative interventions provides unique opportunities for individuals, especially at younger ages, to participate in rewarding experiences that support motivation, ultimately improving patients’ adherence to training protocols [13]. Despite this growing interest, findings about the effect of VR-based interventions that target postural balance in autistic children are still mixed [11,14]. Some studies reported a beneficial effect of VR on balance. Travers and colleagues described a significant increase in timed one-leg stance performance along with reduced postural sway areas after a 6-week visual biofeedback-based balance training using the Wii balance board and Kinect camera, performed three times a week for 60 min in 29 autistic children [10]. Those findings were then replicated and confirmed by the same research group in a randomized controlled trial (RCT), which included 34 autistic subjects and 28 age-matched non-autistic participants [15]. Abdel Ghafar and others investigated the effectiveness of a 12-week non-immersive VR training introduced through the Nintendo Wii Balance Board and Wii Fit Plus combined with traditional physiotherapy versus traditional physiotherapy alone on balance performance in 53 autistic children, finding significant improvement in the overall sway index for the VR training group [16]. Lastly, Caldani and colleagues designed an RCT to explore the effect of a short VR-based training (a single 6-minute postural session consisting of two distinct postural control training exercises using the Balance Quest from Framiral) on postural balance performance of 40 autistic children, reporting a reduction of the sway velocity [17].

Other studies have conversely reported inconclusive findings. In the study of Hocking and colleagues [18], 10 children and adolescents (10–17 years) completed six 20-min VR-based motor training sessions over 2 weeks. Pre-post training analysis on motor skills (assessed with the Bruininks–Oseretsky Test of Motor Proficiency), social, and cognitive outcome measures yielded no significant results. Rafiei Milajerdi and colleagues [19] compared the effects of two types of interventions—exergaming with the Kinect system and the Sports and the Play and Active Recreation for Kids (SPARK)—on motor skills (as measured by the Movement Assessment Battery for Children-2, MABC-2) and executive functions of 60 autistic children. Their results showed an improvement on MABC-2 aiming/catching scores only for participants who underwent SPARK training. Discrepancies between those studies could be due to several reasons, including between-training differences in level of immersiveness, the nature of outcome measures, and the focus on near/far transfer.

To date, no studies have investigated the effect of VR-based training on balance skills of autistic children by using different measures, such as standardized motor tests, caregiver reports, or experimental balance measures. With this respect, previous works from our group highlighted how different sources of information are more effective in describing subtle motor atypicalities in autism [20,21]. Furthermore, previous studies did not explore potential modifications in the walking pattern of autistic children after a VR-based postural balance intervention. Most studies focus on non-immersive VR (using tablet or computer screen), limiting the carry-over to real-life. Immersive VR applications (e.g., a real-life-like visual surround) facilitate training of real-life tasks that children experience as difficult but within a fun, safe and adaptable environment. Given these premises, the aim of the present study is to investigate the effects of a 5-week, immersive VR biofeedback-based training administered with the Gait Real-time Analysis Interactive Lab (GRAIL, Motek, Houten, The Netherlands) system on postural balance of autistic children by means of a multimodal set of outcome measures, which also includes gait features. The GRAIL system combines treadmill training with immersive VR environments and motion capture analysis to collect real-time data and provide visual, auditory and proprioceptive feedback during comprehensive gait and balance training. Furthermore, it allows the assessment of gait and movement strategies in autistic children as demonstrated in two previous studies [22,23]. These studies showed that the GRAIL environment could provide a highly stimulating and rewarding setting for these participants. However, to the best of our knowledge, no studies have yet used this innovative integrated system for postural balance training purposes in autistic children.

Based on previous research, we hypothesized that the present immersive VR biofeedback-based training would result in significant improvements of postural balance of autistic children aged between 6 and 13 years as measured through the GRAIL platform before and after training, whereas we expected a smaller effect on overall motor proficiency, as assessed by standardized measures.

## 2. Materials and Methods

### 2.1. Study Design

**Figure 1:**
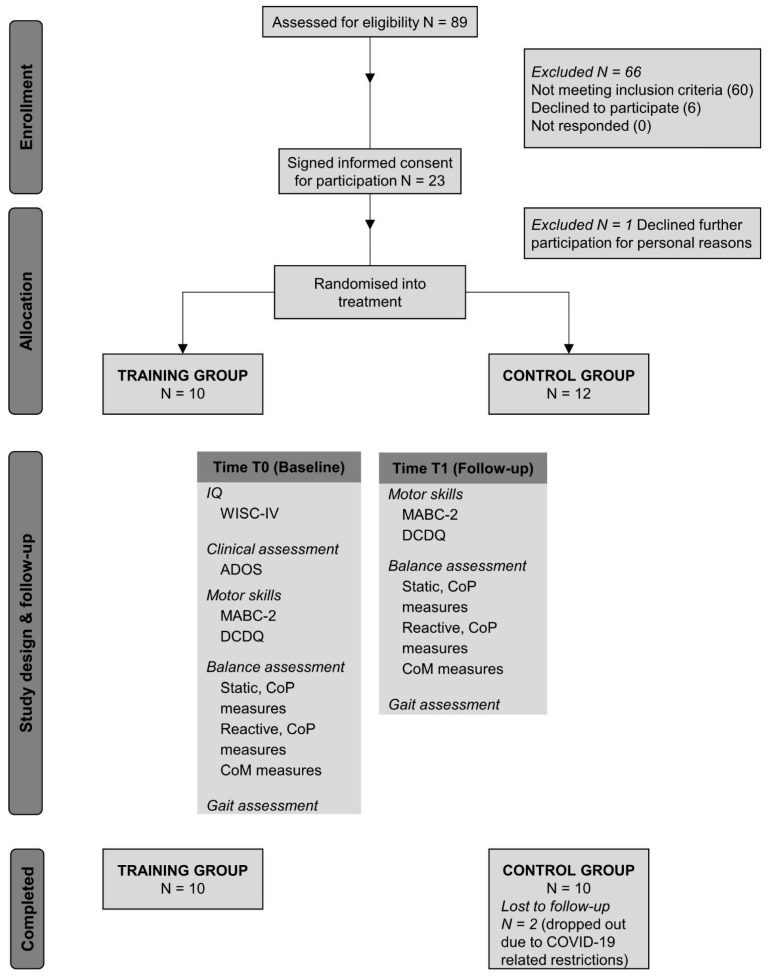
Schematic overview of the study design, with a list of measures collected at each time point. Abbreviations: IQ = Intelligence quotient; WISC-IV = Wechsler Intelligence Scale for Children; ADOS = Autism Diagnostic Observation Schedule; MABC-2 = Movement Assessment Battery for Children-2; DCDQ = Developmental Coordination Disorder Questionnaire; CoP = Center of pressure; CoM = Center of mass.

The present work is a 5-week, randomized, parallel-group, open-label, controlled pilot interventional trial (RCT) investigating the effects of immersive virtual reality-based training administered with the GRAIL (Motek, Houten, The Netherlands) targeting postural balance in autistic children. The trial was registered at ClinicalTrials.gov (identifier: NCT04276571). The study’s reporting complies with the Consolidated Standards of Reporting Trials 2010 statement (see Supplementary Materials). The Ethics Committee of our Institute approved the present study, “Comitato Etico IRCCS E. Medea—Sezione Scientifica Associazione La Nostra Famiglia” (protocol code: Prot. N. 44/19-CE), which was therefore performed following the ethical standards outlined in the 1964 Declaration of Helsinki and later amendments. Prior to participation, written informed consent and oral assent were collected from all caregivers and participants, respectively. Data collection began in October 2019 and ended in August 2022. Figure 1 displays a schematic outline of the design of the present study, including all the measures collected and the flows of participants through the study.

### 2.2. Participants

Participants aged 6 to 13 were recruited from the Child Psychopathology Unit of the Scientific Institute, IRCCS Eugenio Medea (Bosisio Parini, Italy), over a 34-month period.

In order to be eligible for the study, autistic children were required to have an estimated full-scale (FS) intelligence quotient (IQ) of 80 or above, or in cases where the FSIQ was less than 80, subjects were still included if their perceptual reasoning index (PRI) was higher than 80. Indeed, PRI may better estimate cognitive abilities in subjects with autism than the FSIQ, as it is a measure of nonverbal abilities [24].

Participants were excluded in the case that a well-defined genetic disorder was detected. Further exclusion criteria were the use of medication affecting the central nervous system, the presence of significant sensory impairment (e.g., blindness, deafness), abnormalities detected by magnetic resonance imaging (MRI) including epilepsy, and suffering from chronic or acute medical illness. All participants were drug-naïve. After screening for inclusion/exclusion criteria, the study coordinator contacted 29 parents by phone to invite children to participate in the study protocol. Of these, 23 autistic children (19 males, 4 females) and their parents agreed to participate. All participants had been previously diagnosed at our institute on the basis of a consensus “best estimate” DSM-5 clinical diagnostic process informed by, but not dependent on, scores on the Autism Diagnostic Observation Schedule-Second Edition (ADOS-2) [25].

The present study was designed to detect a change in balance performance of 0.7 (i.e., effect size), a power equal to 80% and alpha equal to 0.05, resulting in 10 participants assigned to each group. Considering potential drop out, we enrolled 23 patients.

### 2.3. Intervention

**Figure 2:**
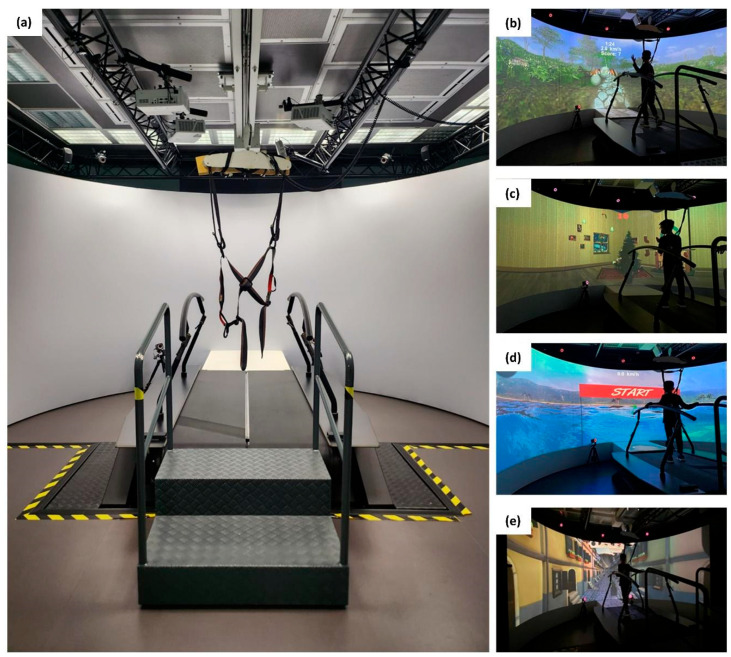
(**a**) The GRAIL system. (**b**–**e**) Examples of exergames played during postural balance training.

Participants were assigned a study number and randomly allocated by an independent third person to either the training or the control group (allocation ratio 1:1) using a computer-generated randomization scheme. Participants in the control group did not perform any treatment, following their normal routine of physical activities. Participants in the training group underwent a virtual reality-based, postural balance training twice a week for a total of 10 sessions (45 min each). This training consisted of various exergames (see Figure 2), selected targeting different postural balance abilities such as encompassing left-right shifting of body weight, monopodalic support, balance maintenance while receiving unexpected external swinging stimuli and dual-task. The protocol was designed to administer each type of exergame on a daily basis, with a total of five exergames per day, with gradually increasing level of difficulty, from the control of the center of mass (CoM) during walking without upper limb support to more challenging tasks, like multitasking activities during walking, external unexpected perturbations (i.e., changes of treadmill slope, single belt sliding, medio-lateral belt sways) and locomotion with decreased step width. The training was performed within the GRAIL system, which is an immersive VR system coupled with motion capture systems to collect real-time data and visual feedback during gait and balance training. It is equipped with an instrumented dual-belt treadmill that integrates 16-channel force plates (sample frequency: 1000 Hz) and is placed over a two-degrees-of-freedom motion frame, which can translate in the longitudinal and lateral direction. Each belt of the treadmill can be independently accelerated or decelerated to assess compensatory strategies and to investigate dynamic stability. The system is surrounded by a motion-capture equipment (Vicon system) with 10 optoelectronic cameras (sample frequency: 100 Hz) to acquire kinematic data and three video cameras. Additionally, a 180° cylindrical projection screen is placed in front of the system where VR environments are projected with an optic flow synchronized to the speed of the treadmill.

### 2.4. Outcomes Measures

At baseline, participants’ general IQ level was evaluated through the Wechsler Intelligence Scale for Children—fourth edition (WISC-IV) [26] to check for eligibility for the study. ADOS-2 total raw scores were converted into calibrated severity scores because the latter are less influenced by participants’ characteristics and allow comparisons across individuals with different developmental levels [27,28].

Furthermore, all participants completed a multimodal evaluation at baseline (Baseline—T0) and after 5 weeks (follow-up—T1).

The a priori primary outcome measures of the study were derived from an experimental balance assessment through the GRAIL platform. This evaluation included a static postural balance assessment through the analysis of the center of pressure (CoP) displacement in four different conditions:

- standing with eyes open—wide feet (EOWF);
- standing with eyes (actively) closed—wide feet (ECWF);
- standing with eyes open—narrowed feet (EONF);
- standing with eyes (actively) closed—narrowed feet (ECNF).

In the first two conditions, the children were asked to keep their feet open at a comfortable distance that was measured and maintained throughout the different conditions. Each trial lasted approximately 60 s, during which children were asked to stand still on one of the two GRAIL treadmill-belt keeping their arms along their sides throughout the test. CoP medio-lateral (ML) and antero-posterior (AP) components were extracted from the GRAIL system. The offset, which corresponds to the initial position of the subject, was then removed subtracting from the ML and AP signals their own means to allow comparison between subjects. Both the ML and AP components were then filtered with a fourth-order low-pass Butterworth filter with a cutoff frequency of 3.5 Hz. Finally, a 60-second window extracted from the medial part of the signal was retained for the analysis.

The following metrics were derived:

- the sway path length, that was calculated as follows (Equation (1)):
  (1) $$sway\;path\;length=\sum_{n=1}^{N-1}\sqrt{{\left(AP\left(n+1\right)-AP\left(n\right)\right)}^{2}+{\left(ML\left(n+1\right)-ML\left(n\right)\right)}^{2}}$$
- the area of the 95% confidence ellipse (hereafter: area CE), that was computed as follows (Equation (2)):
  (2) $$area\;CE=\pi \;\times \;\chi_{2,0.95}^{2}\;\times \;\sqrt{\lambda_{max}\;\times \;\lambda_{min}}$$
  where $\;\chi_{2,0.95}^{2}$ ≈ 5.991 is the 95th percentile of the chi-squared distribution with 2 degrees of freedom; $\lambda_{max}$ and $\lambda_{min}$ are the eigenvalues of the covariance matrix built on the CoP displacement data in the two directions;
- the mean velocity of the CoP both in the ML and AP directions (hereafter: Vel ML, Vel AP), computed as the derivative of the CoP displacement;
- the jerk metrics, which is the derivative of the CoP acceleration.

Additional primary outcome measures of the study were participants’ motor skills, as assessed through the Movement Assessment Battery for Children-2 (MABC-2) [29]. The MABC-2 is designed for children aged 3–16 years and comprises eight age-specific items divided over three age bands. In this study, participants completed either tasks in age band 2 (designed for age 7–10 years) or age band 3 (designed for age 11–16 years). According to children’s cultural reference, we used the Italian normative data [30] to obtain standard scores from single items. A total and three domain standard scores, addressing manual dexterity, ball skills, and static and dynamic balance, respectively, are then computed by summing the item standard scores belonging to each domain, with higher scores indicating better motor performance. The total and domain scores can be interpreted with a traffic light system: the red zone indicates a severe motor impairment (percentile rank (P) ≤ 5), the orange zone indicates being at risk for a motor impairment (P = 6–16), and the green zone indicates normal motor development (P > 16). Both the total and the domain standard scores were recorded as primary outcome measures.

As secondary outcomes, different measures of motor behavior and functioning were used. The parents completed the Developmental Coordination Disorder Questionnaire (DCDQ) [31], which is a 15-item questionnaire, addressing different subdomains of motor abilities, such as ball skills, complex motor coordination, and fine and general motor skills. For each item, parents rate the children’s degree of motor ability on a 5-point scale, comparing it with peers of the same age. The three subscales of the DCDQ, addressing motor control, fine motor, and general coordination, respectively, contribute to determining a total score, with higher scores meaning better motor functioning.

Static balance was also assessed overground for all the four standing conditions described above, using the G-SENSOR (BTS Bioengineering, Milan, Italy). The G-SENSOR is a wearable inertial sensor for gait and motion analysis that embeds four inertial platforms with Sensor Fusion2 technology and a GPS. The components of the inertial platforms are a 16-bit/axis triaxial accelerometer, a 16-bit/axis triaxial gyroscope, and a 13-bit triaxial magnetometer. The G-SENSOR was placed at the fifth lumbar vertebra (L5) level with an ergonomic belt to ensure freedom of movement. During the balance tests, the G-SENSOR was used to acquire the CoM acceleration signal (sample frequency: 100 Hz) with the sensitivity of the accelerometer set to ±2 g, and the sensitivity of the gyroscope set to ±2000°/s. The CoM acceleration signal was pre-processed with the same pipeline as for the CoP displacement signal.

Moreover, reactive postural balance was measured. Subjects were asked to stand on a single belt and to maintain the feet as stable as possible. Three controlled perturbations were applied by accelerating the belt (acceleration of 6 m/s^2^, maximal speed 0.4 m/s), only for the EOWF condition. After each perturbation, the necessary time was granted for a full recovery of balance. The duration of the test was approximately 90 s. Considering the typology of the perturbation, only the CoP AP component was extracted and pre-processed as described above. For each perturbation, the CoP range displacement during the active perturbation (hereafter: Range_p) and during the following recovery time (hereafter: Range_r), the peak value of CoP, the time to peak (TTP) and the time of recovery (TOR) were derived. The features were then averaged across the three perturbations.

Finally, spatio-temporal (walking speed, stance/swing time, step/stance length, step width, and stride time), kinematic (range of motion-ROM- of ankle, knee, and hip) and kinetic (peak of the power curve of flexion/extension of ankle, knee and hip) gait features were collected and processed in real time by means of the 26-marker Human Body Model II [32] using the GRAIL platform. Real-time filtering was performed with a 2nd order Butterworth filter, with a cutoff frequency equal to 6 Hz. The mean and standard deviation of every gait parameter were computed by acquiring several steps during the trial. The standard deviation of the collected features was assessed as a measure of gait pattern variability.

### 2.5. Statistical Analysis

A one-way univariate analysis of variance (ANOVA) was employed to investigate between-group differences in demographic and clinical characteristics of participants (i.e., age, IQ, ADOS) at baseline, to ascertain the comparability of the two groups. Chi-square test was used to examine between-group differences for the categorical variable sex.

Potential between-group differences on outcome measures after the training were investigated using repeated measure analysis of covariance (ANCOVA), with time points (i.e., T0 and T1) as within-subjects factor, group (i.e., training and control group) as between-subjects factor, and considering age as covariate. For significant time by group interactions, simple main effects were assessed.

Significance level was set at *p* < 0.05 for all the analyses. No correction was applied for family wise error rate, as comparisons were strictly planned before the study’s initiation. All statistical analysis was performed with SPSS statistics (version 21, Chicago, IL, USA).

## 3. Results

One child refused to further participate for personal reasons after giving assent and signing the written informed consent. After allocation to training or control groups, two participants, both in the control group, dropped out due to COVID-19 related restrictions (see Figure 1). The final sample therefore resulted in 20 children with autism, assigned to the training (10 participants, 9 males) or control group (10 participants, 8 males), who completed the trial. Demographic and clinical characteristics of the two groups at baseline are reported in Table 1. Chi-square test showed no significant differences in sex between groups (χ^2^ =0.392, *p* = 0.531).

The groups were balanced on IQ, and ADOS, while a marginally significant difference between groups was reported in age, which was higher in the control group. Age was therefore used as covariate in further analyses.

### 3.1. Primary Outcomes

#### 3.1.1. Static Balance Assessment

CoP metrics were not available at T0 and T1 for two participants in the training group because of technical problems during the data acquisition. Table 2 reports descriptive COP metrics values and related statistical results for all four conditions for 10 subjects in the control group and 8 subjects in the training group.

**Figure 3:**
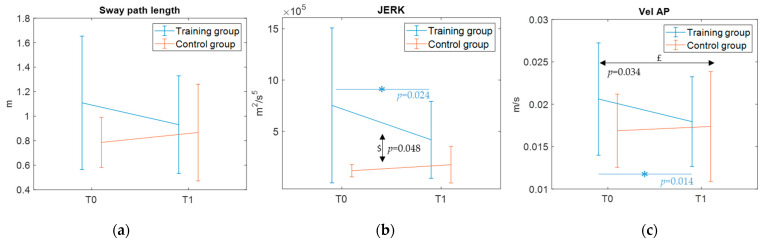
CoP outcomes with significant time by group interaction. (**a**) Sway path length in the EOWF condition. (**b**) Jerk metric in the EONF condition. (**c**) Mean velocity of CoP in the AP direction in the ECNF condition. Mean and standard deviation values are reported. Significant (*p* < 0.05) simple time effects from pairwise comparison are highlighted with an asterisk (and the *p* value) colored as the related group. $: significant main group effects; £: significant main time effects. Abbreviations: AP = antero-posterior component.

The analysis of the CoP sway path length yielded a significant time by group interaction (η^2^_partial_ = 0.237) in the EOWF condition, with participants in the training group showing a decrease in the sway path length. The same trend, although only marginally significant, was observed in the ECWF condition. However, no significant simple time effect was found within each group. A significant time by group interaction was also found for the mean velocity in the AP direction (η^2^_partial_ = 0.328) in the ECNF condition (and marginally significant in the EOWF condition), with participants in the training group decreasing their mean velocity (significant simple time effect in the post hoc pairwise comparison, *p* = 0.014 η^2^_partial_ = 0.338). To note, in the ECNF condition a significant main time effect irrespective of group was also found (η^2^_partial_ = 0.266). Furthermore, the jerk metrics showed significant (η^2^_partial_ = 0.257 in the EONF condition) time by group interactions in all conditions except for the ECNF, with the training group decreasing the mean jerk value (significant simple time effect in the post hoc pairwise comparison for the EONF condition, *p* = 0.024 η^2^_partial_ = 0.296). To note, significant main group effects were also found in EOWF, EONF, and ECWF conditions (η^2^_partial_ = 0.247, 0.237, 0.3, respectively). Figure 3 depicts results for parameters with significant time by group interactions.

No significant effects were found for the area CE and the mean velocity in the ML direction.

#### 3.1.2. MABC-2 Results

**Figure 4:**
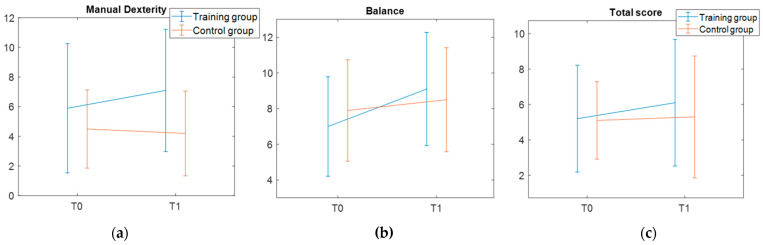
MABC-2 results. (**a**) Manual dexterity domain; (**b**) balance domain; (**c**) total score. Mean and standard deviation values are reported.

With respect to the additional primary outcome measure, MABC-2 data were available for the entire sample of 20 children both at T0 and T1. The analysis did not reveal significant effects of time or group, nor time by group interaction in any MABC-2 measures (Table 3, Figure 4). We additionally explored how participants were distributed in the risk zones according to the traffic light system of MABC-2 (see Supplementary Table S1). Overall, half of them showed severe motor difficulties in the MABC-2 total score, with an equal distribution between groups. Specifically, severe impairments in Manual Dexterity and Aiming and Catching subscales were found in both groups for, respectively, 60% and 40% of participants. Conversely, severe impairments in Balance subscale were found only for 20% of participants in the training and 10% of participants in the control group.

### 3.2. Secondary Outcomes

#### 3.2.1. DCDQ Results

DCDQ data were available for all participants at T0, while at T1 data from one participant in the training group were missing because his/her parents did not complete the questionnaire.

Descriptive data along with the results of the statistical analyses are reported in Table 4. No significant effects were found in any subscale or in the total score.

#### 3.2.2. Static Balance Assessment Overground

**Figure 5:**
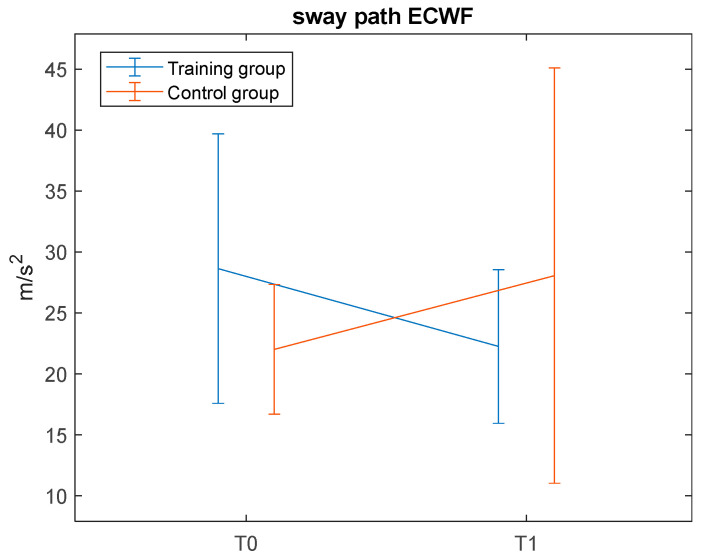
Significant time by group interaction for the COM sway path in the ECWF condition. Abbreviation: ECWF = eyes closed—wide feet.

CoM metrics collected with the G-SENSOR were available for the entire sample of 20 children both at T0 and T1. Analysis of these metrics yielded no significant results, except for the sway path (Table 5), which showed a significant time by group interaction (η^2^_partial_ = 0.258) during the ECWF, in the direction of a decreased sway path in the training group, opposite to the trend of the control group (Figure 5). No significant simple time effect was found in either of the two groups.

#### 3.2.3. Reactive Postural Balance Assessment

CoP displacement data collection was performed on seven subjects for the training group and nine subjects for the control group, because of technical problems during the data acquisition. Statistical analyses yielded no significant differences (Table 6).

#### 3.2.4. Gait Outcomes

**Figure 6:**
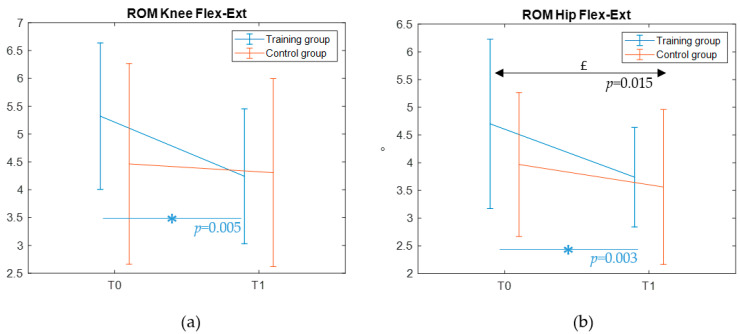
Significant time by group interaction for the variability of the ROM in the knee (**a**) and hip (**b**). Mean and standard deviation values are reported. Significant (*p* < 0.05) simple time effects from pairwise comparison are highlighted with an asterisk (and the *p* value) colored as the related group (blue for training). £: significant main time effects.

Gait features were available for the entire sample of 20 children both at T0 and T1. Table 7 reports descriptive statistics and results of the statistical test performed on gait kinematic parameters in the sagittal plane. Specifically, the mean values and the standard deviations of the ROM of the ankle, knee, and hip are shown. We did not find any significant effect of time or group, nor time–group interaction effects in the mean values of such metrics. Interestingly, the variability of the ROM in the knee highlighted a significant time by group interaction (η^2^_partial_ = 0.233), with a significant time effect in the post hoc pairwise comparison for the training group (*p* = 0.005). Similarly, the variability of the ROM in the hip showed a statistically significant time by group interaction (η^2^_partial_ = 0.236), with a significant time effect in the post hoc pairwise comparison for the training group (*p* = 0.003) (Figure 6). Moreover, a significant time effect, irrespective of group, was observed in the ROM of the hip (η^2^_partial_ =0.3) with a greater decrease in the training group with respect to the control one.

No significant results were found for the spatio-temporal and kinetic parameters, which are presented in the Supplementary Tables S2 and S3.

**Figure 7:**
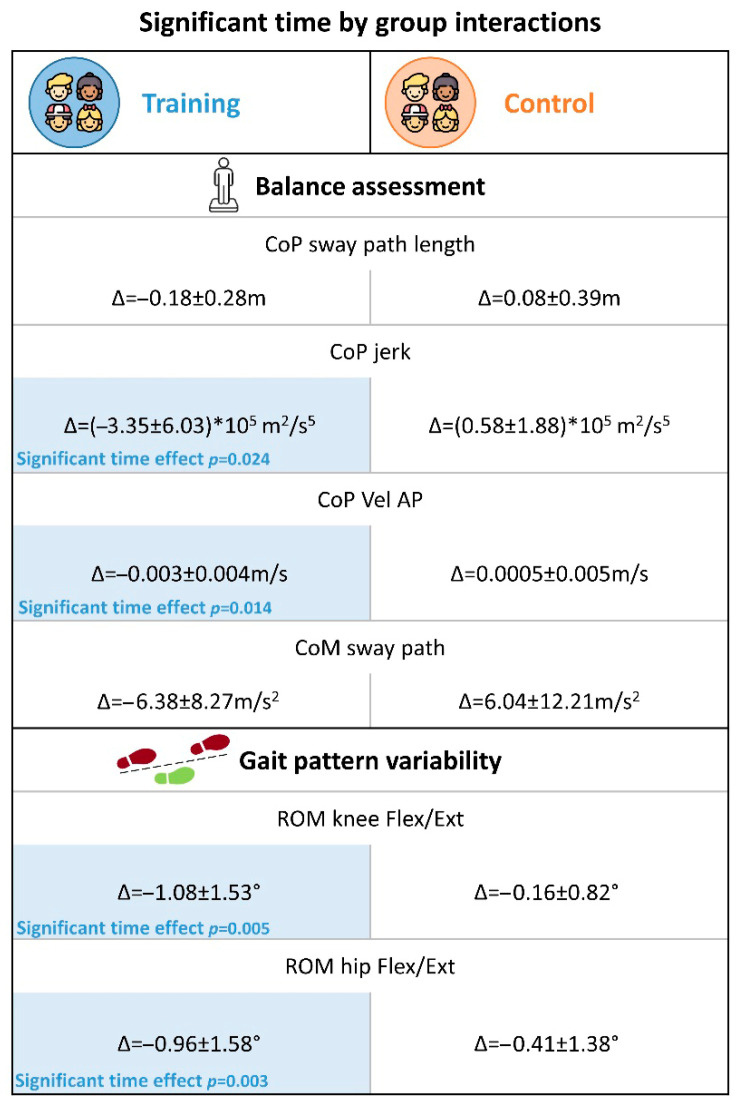
Key findings of the present study. Pre-post intervention differences for those outcomes that presented significant time by group interactions are reported. Colored cells represent significant time effect given by the post hoc analysis.

Figure 7 presents an overall synthesis of the final data analysis findings, aimed at facilitating clearer and more efficient understanding for the reader.

## 4. Discussion

The objective of the present clinical trial was to investigate the effects of a 5-week, immersive VR biofeedback-based training on postural balance of autistic children. Significant improvements were expected in postural balance metrics, whereas smaller effect on overall motor proficiency was hypothesized. To the best of our knowledge, this pilot study is one of the first attempts to perform a multimodal analysis combining caregiver reports, standardized motor test, static and reactive postural balance assessments, and gait metrics, by leveraging the innovative integrated GRAIL system. Among its multiple features, the intervention here proposed specifically benefited from the visual, proprioceptive, and auditory feedback provided to enhance the participant’s motivation while supporting the training. Furthermore, the combination of VR environments, treadmill use and motion capture technology enabled both static and reactive postural balance assessments, as well as gait pattern analysis.

With regard to the first primary outcome—a static balance assessment through the GRAIL platform—the present results indicated slight yet significant time by group interactions for the CoP measures sway path length, AP-velocity, and jerk metrics, with the training group demonstrating a trend of improvement in postural stability. To note, the results also indicate a time effect for AP-velocity. Thus, this improvement could be likely due to a mixed effect of training intervention but also of test–retest familiarity. Nonetheless, these parameters are overall critical indicators of balance abilities [33]. Shorter sway paths reflect reduced overall body sway proving enhanced stability, lower AP-velocities indicate a reduced muscular effort required to improve postural control [34], and reduced jerks suggest more coordinated postural adjustments. These findings are corroborated by the significant time by group interaction observed for CoM—a secondary outcome measure—in the ECWF condition only, where the training group showed a decrease in sway path. These significant interactions, along with the significant time effects found in the post hoc comparison for the training group only (see Figure 7), may suggest that the differences over time between groups may be training-induced. Despite significant methodological differences, the present results are in line with those reported by previous well-powered studies [10,15,16,17]. However, factors such as the intensity and frequency of the training may have critically limited the effect size of the present immersive VR training on postural balance performance. In this respect, it is worth acknowledging that participants of Travers et al. and Abdel Ghafar et al., underwent training three times a week for 6 weeks and 12 weeks, respectively, whereas participants in the present study underwent training twice a week for 5 weeks. Future extensions of this trial should therefore consider increasing both the intensity and the duration of the training to obtain potentially more robust findings.

As for the second primary outcome—the assessment of motor skills with the MABC-2—the results did not show any significant training effect. While this negative finding aligns with other studies, which used standardized motor tests to assess treatment-induced changes [18,19], it is also important to consider that both the present study and the Rafiei Milajerdi and colleagues’ one detected after-training differences in the experimental measures of balance. Findings from those two studies therefore provide converging evidence that existing standardized motor batteries are not necessarily sensitive to little modifications over a short period of time within a single domain (i.e., balance). On one hand, this could be related to the psychometric properties or the nature of the instrument itself—Bruininks–Oseretsky Test and MABC-2 are used to identify significant delays at motor skills level—whereas validated tests that functionally assess postural balance succeed in detecting significant training effect (see, for example, the Pediatric Balance Scale used by [16]). Alternatively, it is plausible that functional ameliorations in postural balance induced by the VR training programs are too limited to induce modifications at the motor skills level.

Concerning the secondary outcome variables, no statistically significant results were found in the reactive postural balance assessment performed with GRAIL. This null finding differed from the significant after-training changes reported by Caldani and others and Abdel Ghafar and others [16,17]. However, those previous studies estimated the training-related changes in dynamic balance by measuring participants’ body sways while standing on an unstable platform, whereas we recorded the COP displacement after three discrete treadmill perturbations.

No significant changes were observed in gait features after training either. However, a significant reduction of the gait pattern variability was found for the ROM of the knee and hip during flexion in the training group, suggesting that although the treatment may not induce major changes in kinematics, it contributes to a more stable gait pattern. To date, no studies have yet explored potential modifications in the walking pattern of autistic children after a VR-based postural intervention. The absence of comparable prior evidence hinders our ability to disentangle the underlying reasons for the present findings. On one hand, it is possible that the lack of a training impact on gait could reflect a ceiling effect—as also observed for the MABC-2 performance range on the balance subscale—with autistic participants enrolled in this study not showing atypicalities in the spatio-temporal and kinematic features of gait (in line with previous comparisons with typically developing peers, see [22]). More broadly, this result could be due to a general dose-effect, as suggested by the meta-analysis of [35]. In fact, an intervention of greater duration or intensity may be required to observe substantial changes. In contrast, the intervention planned for the training group in our study amounted to 7.5 h, which is notably below the 16-hour threshold identified by [35] as necessary to achieve a significant effect on gross motor outcomes. Future extensions of this trial should therefore consider moving in this direction to obtain potentially more robust findings.

Lastly, no training-induced differences were observed in caregivers’ reports of motor skills. As for the MABC-2 results, this negative finding could be related to the content of the questionnaire itself, which did not include specific questions about postural balance skills.

The present findings should be interpreted in light of several limitations. First, this study was limited by its small sample size, likely due to an overestimation of the expected effect size. Despite being a pilot study, the present trial found limited but significant evidence of VR biofeedback-based training effectiveness. However, a larger sample size might have revealed additional benefits. These preliminary results therefore need to be replicated in a larger, independent sample. A further limitation is that participants were not selected based on their motor proficiency, which could have helped target the intervention specifically to children with documented motor, particularly balance, difficulties. Notably, only 15% of participants exhibited balance difficulties. Nevertheless, the study detected small but significant improvements in balance within the training group. Conversely, our unbiased selection included a sample of autistic children with average balance skills (see Supplementary Table S1), potentially restraining the present findings. Moreover, data related to body mass index of the participants were not collected and the present results were therefore not controlled for this potential moderator. Lastly, we acknowledge that the degree of immersion provided by the GRAIL system may be lower compared to other IVR systems, such as helmets or goggles. However, we do not believe this limitation affects our results, as previous studies using non-immersive VR systems have reported significant and robust findings (e.g., [16]). Due to the novelty of the present approach, future extensions of this study are needed to ascertain the generalizability of the present findings, also considering the motor profile.

## 5. Conclusions

The present study evaluated the effectiveness of IVR postural balance training performed with the GRAIL system in autistic children by means of multimodal analysis. Observations derived from the comparison of caregiver reports, standardized motor tests, postural balance and gait outcomes of the training group with those of the control one (who followed normal routine of physical activities) suggest that differences over time between groups may be training-induced, as significant improvements over time can be observed only in the training group. Despite some limitations, such as the small sample size and low training intensity, this pilot work contributes to the understanding of the effects VR biofeedback-based training on postural balance in autism, taking into account also potential modifications in the walking pattern. This could pave the way for future multimodal approaches that could give a more comprehensive assessment of related interventions.

## Figures and Tables

**Table 1:** Participants’ demographic and clinical characteristics at baseline along with one-way ANOVA results. Italic text represents significance at a trend level (*p* < 0.07).

	Group	N	Mean	SD	F	*p* Value
age	training	10	9.14	1.85	*4.075*	*0.059*
	control	10	10.75	1.71		
IQ	training	10	97.9	15.42	0.203	0.658
	control	9	101.44	18.82		
ADOS	training	6	5.33	2.34	1.411	0.256
	control	9	6.56	1.67		

**Table 2:** CoP outcomes. Mean and standard deviation (SD) values are reported. Bold text represents statistically significant differences (*p* < 0.05), while italic text represents moderately significant differences (*p* < 0.07). Abbreviations: EOWF = eyes open—wide feet; ECWF = eyes closed—wide feet; EONF = eyes open—narrowed feet; ECNF = eyes closed—narrowed feet; area CE = area of the 95% confidence ellipse; Vel AP = mean velocity in the antero-posterior direction; Vel ML = mean velocity in the medio-lateral direction.

			T0	T1	Time Effect		Group Effect		Time–Group Interaction	
	CoP Metrics	Group	Mean (SD)	Mean (SD)	F	*p*	F	*p*	F	*p*
EOWF	sway path length (m)	training	1.109 (0.544)	0.931 (0.398)	2.406	0.142	0.056	0.816	**4.65**	**0.048**
		control	0.786 (0.204)	0.867 (0.394)						
	area CE (mm^2^)	training	0.615 (0.71)	0.744 (0.863)	1.591	0.226	0.222	0.644	0.073	0.791
		control	0.904 (1.493)	0.574 (0.632)						
	Vel AP (m/s)	training	0.014 (0.007)	0.011 (0.004)	0.719	0.410	0.349	0.563	*4.492*	*0.051*
		control	0.01 (0.002)	0.01 (0.004)						
	Vel ML (m/s)	training	0.009 (0.005)	0.009 (0.004)	*4.220*	*0.058*	0.052	0.822	2.452	0.138
		control	0.007 (0.003)	0.008 (0.005)						
	Jerk (m^2^/s^5^)	training	574,672.12 (598,397.92)	234,334.86 (176,189.63)	0.102	0.754	**4.922**	**0.042**	*3.858*	*0.068*
		control	103,342.19 (71,850.74)	146,417.44 (118,914.29)						
EONF	sway path length (m)	training	1.481 (0.452)	1.417 (0.505)	0.493	0.493	0.882	0.363	0.028	0.869
		control	1.216 (0.278)	1.122 (0.41)						
	area CE (mm^2^)	training	1.131 (0.519)	1.265 (0.759)	1.124	0.306	0.100	0.756	0.055	0.818
		control	1.287 (1.06)	1.139 (0.885)						
	Vel AP (m/s)	training	0.018 (0.006)	0.017 (0.006)	2.274	0.152	1.042	0.323	0.506	0.488
		control	0.014 (0.003)	0.013 (0.006)						
	Vel ML (m/s)	training	0.013 (0.004)	0.014 (0.005)	0.016	0.901	0.930	0.350	1.046	0.323
		control	0.012 (0.003)	0.011 (0.003)						
	Jerk (m^2^/s^5^)	training	753,011.65 (754,781.15)	418,082.328 (374,255.77)	0.851	0.371	**4.653**	**0.048**	**5.192**	**0.038**
		control	116,838.17 (59,996.94)	174,949.414 (177,678.23)						
ECWF	sway path length (m)	training	1.277 (0.547)	1.11 (0.341)	0.431	0.521	0.283	0.602	*4.441*	*0.052*
		control	0.939 (0.157)	1.044 (0.365)						
	area CE (mm^2^)	training	0.726 (0.749)	0.758 (0.63)	0.831	0.376	0.337	0.570	2.128	0.165
		control	0.47 (0.351)	1.085 (1.35)						
	Vel AP (m/s)	training	0.016 (0.007)	0.014 (0.004)	0.979	0.338	0.317	0.582	3.582	0.078
		control	0.012 (0.002)	0.013 (0.004)						
	Vel ML (m/s)	training	0.011 (0.005)	0.01 (0.004)	0.033	0.857	0.252	0.623	1.265	0.278
		control	0.008 (0.002)	0.009 (0.004)						
	Jerk (m^2^/s^5^)	training	650,637.707 (670,198.2)	272,446.434 (189,326.68)	0.280	0.604	**6.420**	**0.023**	*4.528*	*0.050*
		control	92,588.638 (51,433.35)	153,904.579 (88,378.67)						
ECNF	sway path length (m)	training	1.705 (0.502)	1.538 (0.465)	2.635	0.125	0.020	0.889	2.035	0.174
		control	1.451 (0.348)	1.399 (0.489)						
	area CE (mm^2^)	training	1.168 (0.665)	1.417 (0.661)	0.640	0.436	0.042	0.841	0.131	0.722
		control	1.24 (0.82)	1.157 (1.085)						
	Vel AP (m/s)	training	0.021 (0.007)	0.018 (0.005)	**5.431**	**0.034**	0.002	0.965	**7.307**	**0.016**
		control	0.017 (0.004)	0.017 (0.006)						
	Vel ML (m/s)	training	0.015 (0.004)	0.015 (0.005)	0.588	0.455	0.129	0.724	0.041	0.843
		control	0.014 (0.004)	0.012 (0.004)						
	Jerk (m^2^/s^5^)	training	836,076.711 (837,155.07)	382,124.895 (293,048.1)	0.189	0.670	3.618	0.077	3.441	0.083
		control	214,674.2 (162,150.59)	224,108.55 (180,316.57)						

**Table 3:** MABC-2 domain and total scores. Mean and standard deviation (SD) values are reported.

		T0	T1	Time Effect		Group Effect		Time–Group Interaction	
MABC-2	Group	Mean (SD)	Mean (SD)	F	*p*	F	*p*	F	*p*
Manual Dexterity	training	5.9 (4.358)	7.1 (4.122)	0.000	0.988	0.888	0.359	0.807	0.381
	control	4.5 (2.635)	4.2 (2.86)						
Aiming and Catching	training	6.2 (3.994)	5.5 (3.979)	0.003	0.955	1.081	0.313	1.011	0.329
	control	6.4 (3.777)	7.6 (4.671)						
Balance	training	7 (2.789)	9.1 (3.178)	0.629	0.439	0.376	0.548	0.296	0.594
	control	7.9 (2.846)	8.5 (2.915)						
Total	training	5.2 (3.011)	6.1 (3.573)	0.280	0.604	0.105	0.750	0.141	0.712
	control	5.1 (2.183)	5.3 (3.433)						

**Table 4:** DCDQ subscales and total scores. Mean and standard deviation (SD) values are reported.

		T0	T1	Time Effect		Group Effect		Time–Group Interaction	
DCDQ Item	Group	Mean (SD)	Mean (SD)	F	*p*	F	*p*	F	*p*
Control	training	16 (4.873)	16.889 (4.512)	0.015	0.906	3.181	0.093	0.338	0.569
	control	19.1 (6.855)	21.5 (6.346)						
Fine motor	training	11.556 (2.455)	11.667 (3.317)	1.551	0.231	1.414	0.252	2.988	0.103
	control	11.9 (5.043)	13.7 (4.165)						
General coordination	training	12.444 (4.126)	13.222 (4.236)	0.091	0.767	3.841	0.068	0.292	0.596
	control	14.8 (6.546)	16.4 (5.125)						
Total Score	training	40 (10.087)	41.778 (10.485)	0.186	0.672	3.360	0.085	0.985	0.336
	control	45.8 (17.511)	51.6 (14.478)						

**Table 5:** CoM sway path outcomes in the four conditions. Mean and standard deviation (SD) values are reported. Bold text represents statistically significant differences (*p* < 0.05). Abbreviations: EOWF = eyes open—wide feet; ECWF = eyes closed—wide feet; EONF = eyes open—narrowed feet; ECNF = eyes closed—narrowed feet.

		T0	T1	Time Effect		Group Effect		Time–Group Interaction	
CoM Sway Path	Group	Mean (SD)	Mean (SD)	F	*p*	F	*p*	F	*p*
EOWF	training	28.197 (13.566)	22.995 (8.28)	0.227	0.640	0.595	0.451	2.372	0.142
	control	20.972 (6.05)	26.497 (20.107)						
EONF	training	36.441 (13.308)	33.126 (11.092)	2.219	0.155	0.243	0.628	3.036	0.099
	control	30.474 (8.113)	34.676 (20.897)						
ECWF	training	28.638 (11.058)	22.251 (6.304)	0.037	0.850	0.456	0.509	**5.907**	**0.026**
	control	22.015 (5.319)	28.060 (17.036)						
ECNF	training	38.049 (13.87)	30.209 (9.821)	0.163	0.691	0.893	0.358	1.942	0.181
	control	33.776 (8.631)	33.889 (17.416)						

**Table 6:** CoP outcomes for the reactive postural balance assessment. Mean and standard deviation (SD) values are reported. Abbreviations: Range_p = range displacement during the active perturbation; Range_r = range displacement during the recovery period; TTP = time to peak; TOR = time of recovery.

		T0	T1	Time Effect		Group Effect		Time–Group Interaction	
CoP Metrics	Group	Mean (SD)	Mean (SD)	F	*p*	F	*p*	F	*p*
Range_p (m)	training	0.113 (0.017)	0.107 (0.008)	1.566	0.233	1.146	0.304	0.018	0.896
	control	0.123 (0.021)	0.107 (0.017)						
Range_r (m)	training	0.051 (0.037)	0.045 (0.015)	0.256	0.621	0.782	0.393	0.039	0.846
	control	0.045 (0.015)	0.039 (0.019)						
Max (m)	training	0.03 (0.016)	0.026 (0.006)	0.027	0.873	0.393	0.542	0.002	0.962
	control	0.029 (0.008)	0.023 (0.01)						
TTP (s)	training	9.516 (6.735)	14.484 (9.45)	0.006	0.939	0.713	0.414	0.003	0.961
	control	7.822 (5.247)	13.372 (8.372)						
TOR (s)	training	3.722 (2.119)	3.438 (1.182)	0.059	0.812	0.021	0.888	0.241	0.632
	control	5.272 (5.01)	3.031 (1.456)						

**Table 7:** Gait kinematic metrics. Mean and standard deviation (SD) values are reported. Bold text represents statistically significant differences (*p* < 0.05). Abbreviations: ROM = range of motion; Flex-Ext = flexion-extension.

			T0	T1	Time Effect		Group Effect		Time–Group Interaction	
	Gait Features	Group	Mean (SD)	Mean (SD)	F	*p*	F	*p*	F	*p*
Mean values	ROM Ankle Flex-Ext (°)	training	20.4 (3.62)	19.7 (2)	0.694	0.416	0.118	0.736	2.425	0.138
		control	18.46 (2.5)	19.48 (3.57)						
	ROM Knee Flex-Ext (°)	training	63.07 (4.26)	64.27 (5.18)	0.111	0.743	0.020	0.888	1.678	0.213
		control	63.12 (4.41)	61.79 (4.07)						
	ROM Hip Flex-Ext (°)	training	37.2 (4.42)	38.46 (2.87)	0.241	0.630	0.929	0.349	1.110	0.307
		control	39.28 (3.43)	38.06 (2.79)						
Standard deviation	ROM Ankle Flex-Ext (°)	training	3.52 (1.84)	2.81 (0.78)	0.530	0.476	0.328	0.574	2.619	0.124
		control	3.22 (1)	3.32 (1.02)						
	ROM Knee Flex-Ext (°)	training	5.32 (1.31)	4.24 (1.21)	1.398	0.253	0.039	0.846	**5.154**	**0.036**
		control	4.46 (1.8)	4.31 (1.69)						
	ROM Hip Flex-Ext (°)	training	4.7 (1.53)	3.74 (0.9)	**7.295**	**0.015**	0.388	0.542	**5.255**	**0.035**
		control	3.97 (1.3)	3.56 (1.4)						

## Data Availability

The original data presented in the study are deposited on Zenodo and openly available as of the date of publication at https://doi.org/10.5281/zenodo.15781767 (accessed on 2 July 2025).

## Supplementary Materials

The following supporting information can be downloaded at: https://www.mdpi.com/article/10.3390/jcm14165616/s1, Table S1: Classification of participants at T0 in each risk class (green, amber, or red zone) according to the MABC-2 traffic light system classification, reported for each subscale and for the total MABC-2 score; Table S2: Gait spatio-temporal and kinetic mean values metrics. Mean and standard deviation (SD) values are reported; Table S3: Gait spatio-temporal and kinetic standard deviation metrics. Mean and standard deviation (SD) values are reported.

## Abbreviations

The following abbreviations are used in this manuscript:

VR	Virtual Reality
RCT	Randomized Controlled Trial
SPARK	Sports and the Play and Active Recreation for Kids
MABC-2	Movement Assessment Battery For Children-2
GRAIL	Gait Real-Time Analysis Interactive Lab
FSIQ	full-scale intelligence quotient
PRI	perceptual reasoning index
MRI	Magnetic Resonance Imaging
ADOS-2	Autism Diagnostic Observation Schedule-Second Edition
CoM	Center Of Mass
WISC-IV	Wechsler Intelligence Scale For Children—Fourth Edition
CoP	Center Of Pressure
EOWF	Eyes Open—Wide Feet
ECWF	Eyes Closed—Wide Feet
EONF	Eyes Open—Narrowed Feet
ECNF	Eyes Closed—Narrowed Feet
ML	Medio-Lateral
AP	Antero-Posterior
area CE	Area Of The 95% Confidence Ellipse
DCDQ	Developmental Coordination Disorder Questionnaire
TTP	Time To Peak
TOR	Time Of Recovery
ROM	Range of motion
ANCOVA	Analysis Of Covariance
SD	Standard Deviation
